# Molecular characterization of carbapenem-resistant and virulent plasmids in *Klebsiella pneumoniae* from patients with bloodstream infections in China

**DOI:** 10.1080/22221751.2021.1906163

**Published:** 2021-04-05

**Authors:** Yongqiang Yang, Yanxian Yang, Guanping Chen, Minmin Lin, Yuan Chen, Ruowen He, Klibs N. Galvão, Mohamed Abd El-Gawad El-Sayed Ahmed, Adam P. Roberts, Yiping Wu, Lan-Lan Zhong, Xiaoxue Liang, Mingyang Qin, Xin Ding, Wenbin Deng, Songyin Huang, Hong-Yu Li, Min Dai, Ding-Qiang Chen, Liyan Zhang, Kang Liao, Yong Xia, Guo-Bao Tian

**Affiliations:** a Department of Microbiology, Zhongshan School of Medicine, Sun Yat-sen University, Guangzhou, People’s Republic of China; b Key Laboratory of Tropical Diseases Control (Sun Yat-sen University), Ministry of Education, Guangzhou, People’s Republic of China; c School of Pharmaceutical Sciences (Shenzhen), Sun Yat-sen University, Guangzhou, People’s Republic of China; d Sun Yat-sen University School of Medicine, Guangzhou, People’s Republic of China; e Department of Respiratory Medicine, the Fifth Affiliated Hospital of Sun Yat-sen University, Zhuhai, People’s Republic of China; f Department of Large Animal Clinical Sciences, College of Veterinary Medicine, University of Florida, Gainesville, FL, USA; g Department of Microbiology and Immunology, Faculty of Pharmaceutical Sciences and Drug Manufacturing, Misr University for Science and Technology, Cairo, Egypt; h Department of Tropical Disease Biology, Liverpool School of Tropical Medicine, Pembroke Place, UK; i Centre for Drugs and Diagnostics, Liverpool School of Tropical Medicine, Pembroke Place, UK; j School of Laboratory Medicine, Chengdu Medical College, Chengdu, People’s Republic of China; k Basic Medical College, Xinxiang Medical University, Xinxiang, People’s Republic of China; l Department of Clinical Laboratory, Sun Yat-sen Memorial Hospital, Sun Yat-sen University, Guangzhou, People’s Republic of China; m Division of Laboratory Medicine, Zhujiang Hospital, Southern Medical University, Guangzhou, People’s Republic of China; n Department of Clinical Laboratory, Guangdong Provincial People’s Hospital / Guangdong Academy of Medical Sciences, Guangzhou, People’s Republic of China; o Department of Clinical Laboratory, the First Affiliated Hospital of Sun Yat-Sen University, Guangzhou, People’s Republic of China; p Department of Clinical Laboratory Medicine, Third Affiliated Hospital of Guangzhou Medical University, Guangzhou, People’s Republic of China; q School of Medicine, Xizang Minzu University, Xianyang, People’s Republic of China

**Keywords:** Bloodstream infection, carbapenem resistance, *Klebsiella pneumoniae*, genomics, KPC-2

## Abstract

Bloodstream infections (BSIs) caused by carbapenem-resistant *Klebsiella pneumoniae* (CRKP) are potentially life-threatening and an urgent threat to public health. The present study aims to clarify the characteristics of carbapenemase-encoding and virulent plasmids, and their interactions with the host bacterium. A total of 425 *Kp* isolates were collected from the blood of BSI patients from nine Chinese hospitals, between 2005 and 2019. Integrated epidemiological and genomic data showed that ST11 and ST307 *Kp* isolates were associated with nosocomial outbreak and transmission. Comparative analysis of 147 *Kp* genomes and 39 completely assembled chromosomes revealed extensive interruption of *acrR* by IS*Kpn26* in all *Kp* carbapenemase-2 (KPC-2)-producing ST11 *Kp* isolates, leading to activation of the AcrAB-Tolc multidrug efflux pump and a subsequent reduction in susceptibility to the last-resort antibiotic tigecycline and six other antibiotics. We described 29 KPC-2 plasmids showing diverse structures, two virulence plasmids in two KPC-2-producing *Kp*, and two novel multidrug-resistant (MDR)-virulent plasmids. This study revealed a multifactorial impact of KPC-2 plasmid on *Kp*, which may be associated with nosocomial dissemination of MDR isolates.

## Introduction

Bloodstream infections (BSIs) caused by Enterobacterales have become increasingly life-threatening, leading to a mortality rate as high as 48% [1,2]. Carbapenems remain one of the first-line of therapeutic agents for BSIs. Therefore, the emergence of carbapenemase-mediated resistance represents a serious public health threat [3]. Carbapenem resistance has been associated with increased length of hospital stay and mortality of BSI patients [4]. Carbapenemase-encoding plasmids can be transferred among various Enterobacterales via horizontal gene transfer (HGT) and disseminated in hospitals [5]. As a result, carbapenem-resistant Enterobacterales (CRE) have been reported worldwide [6]. *Klebsiella pneumoniae* is a clinically important species and causes serious nosocomial infections such as septicemia, pneumonia, urinary tract infection, surgical site infection, and soft tissue infection [7]. In China, carbapenem-resistant *Klebsiella pneumoniae* (CRKP) accounts for about 64% of CRE infections [8]. Nonetheless, the characteristics of carbapenemase-encoding plasmids and their interactions with the host bacterium are not fully understood.

The hypervirulent variant of *Kp* (HvKp) has been increasingly reported in association with plasmid-mediated virulence loci *rmpA*/*rmpA2*, *iuc*, and *iro* [9,10]. The *rmpA*/*rmpA2* genes encode proteins regulating capsule production in *Kp*, while *iucABCDiutA* and *iroBCDN* are responsible for the biosynthesis of siderophores aerobactin and salmochelin, respectively [11]. Carbapenem-resistant and virulence plasmid-carrying *Kp* is associated with excess morbidity and mortality in China [12]. The emergence and dissemination of carbapenem-resistant HvKp (CR-HvKp) are of great concern due to the combination of virulence and lack of treatment options.

Herein, we conducted integrated epidemiological and genomic analysis to infer the resistomes, virulence determinants, and the phylogenetic relationship between CRKP and CSKP isolates. We demonstrated that IS*Kpn26* insertion contributed to the MDR phenotypes in all the ST11-*bla*_KPC-2_ *Kp* by blocking the expression of AcrAB-TolC repressor *acrR*. Furthermore, we identified novel MDR-virulent plasmids due to ongoing recombination in *Kp*, representing a significant health threat in terms of both disease and treatment.

## Material and methods

### Bacterial isolates

We collected 425 *Kp* isolates from the blood of BSI patients from nine tertiary hospitals in Guangdong province, China, between 2005 and 2019 (Table S1). In cases with multiple positive blood cultures, we only included the first positive blood culture. Preliminary species identification was achieved by MALDI-TOF MS (BrukerDaltonik GmbH, Bremen, Germany) and 16S rRNA sequencing. Ethical approval for this study was given by Zhongshan School of Medicine of Sun Yat-sen University under approval number 068.

### Antimicrobial susceptibility testing, s1-PFGE, and Southern blotting

The minimum inhibitory concentrations (MICs) were determined for 15 antibiotics for all isolates using the agar dilution method with the exception of colistin, which used the broth dilution method following the Clinical and Laboratory Standards Institute (CLSI) guidelines [13]. MIC determinations were also carried out with fixed concentration (100 μg/mL) of the efflux pump inhibitor 1-(1-naphthylmethyl)-piperazine (NMP) against 20 antibiotics among all the ST11-*bla*_KPC-2_ strains. The plasmid location of the carbapenem encoding gene was determined by S1-nuclease digestion and pulsed-field gel electrophoresis (S1-PFGE), followed by Southern blotting hybridizations with a *bla*_KPC-2_ probe [14].

## Galleria mellonella *infection model*

The virulence of target strains was determined using the wax moth (*G. mellonella*) larvae model [12,15]. Three doses of 1×10⁴, 1×10^5^, 1×10^6^ CFU each with ten worms per group were tested. 1×10⁴ CFU was used for the injection. Controls included a PBS injection group, one group receiving no dose, a non-virulent control using *E. coli* MG1655, and a highly-virulent control using HvKP4 as previously reported [12]. The larvae were incubated at 37°C in a darkroom and the survival rate was recorded every 12 h for seven days. The experiments were conducted in duplicate.

### Quantitative real-time PCR (qPCR)

The experimental procedures for qPCR were modified from a previous report [16]. The total RNA of *Kp* strains was extracted using the bacteria RNA Extraction Kit (Vazyme Biotech, China). Reverse transcription was performed using Goldenstar^TM^ RT6 cDNA Synthesis Kit Ver.2 (Beijing TsingKe Biotech, China). The qPCR assay was conducted using the Bio-Rad IQ thermocycler and Master qPCR Mix-SYBR (Beijing TsingKe Biotech, China) for three biological replicates and three technical replicates. Calculation of 2^-ΔΔ^CT using 16S rRNA as the reference was used to determine the relative transcript levels for each target gene of *acrA*, *acrB*, and *acrR*. The primers used to amplify each gene are listed in Table S2.

### Whole-genome sequencing and genotyping

All the 72 CRKP isolates and 82 randomly selected carbapenem-susceptible *Kp* (CSKP) isolates from the contributing hospitals were selected for whole-genome sequencing (WGS). DNA libraries were constructed with 350-bp paired-end fragments and sequenced using an Illumina HiSeq 2000 platform. Short-read sequence data were *de novo* assembled using SPAdes v3.10 [17]. For the reference strains (Table S3), eight ST11 strains and one ST23 strain from China [12], 140 ST11 strains from Europe [18], six ST11 strains from other countries in Asia [19], and six outbreak-associated ST258 strains from the USA were enrolled [20]. Furthermore, to detect the IS*Kpn26* insertion into *acrR* across public strains, all fully assembled *Kp* genomes were downloaded from GenBank as of 1/1/2021. The long-read MinION sequencing (Oxford Nanopore Technologies, Oxford, UK) was used to sequence 40 *Kp* strains out of the 154 newly sequenced strains with a mean read length of 24 kbp. These isolates included all the 34 ST11 strains, two KPC-2-producing non-ST11 strains, and four virulence plasmid-carrying strains. *De novo* hybrid assembly both of short Illumina reads and long MinION reads was performed using Unicycler v0.4.3 [21], and corrected using Pilon v1.22. Plasmid sequences were confirmed by manually extracting the sequences from the assemblies to conduct a BLASTn search. The MLST and cgMLST were identified using the BIGSdb (//bigsdb.web.pasteur.fr/klebsiella/). The minimum spanning tree (MST) was constructed by GrapeTree [22]. Acquired antibiotic resistance genes (ARGs) and virulence genes were identified using ABRicate version 0.5 (https://github.com/tseemann/abricate) by aligning genome sequences to the ResFinder database [23] and VFDB database [24]. IS elements (https://www-isfinder.biotoul.fr), CRISPRs (https://crisprcas.i2bc.paris-saclay.fr/CrisprCasFinder/Index), PAIs (http://www.paidb.re.kr) [25], and prophages (https://phaster.ca/) [26] were identified using web-based searches. Kaptive was used to identify the whole capsule synthesis locus (K-locus) based on assembly scaffolds [27].

### Phylogenetic analysis

For each *de novo* assembly, coding sequences were predicted using Prodigal v2.6 [28] and annotated using Prokka v1.13.3 [29]. Core genes were identified and used to build the core genome using Roary v3.12 [30] with the –e –mafft setting to create a concatenated alignment of core genomic CDS. SNP-sites (https://github.com/sanger-pathogens/snp-sites) was used to extract the core-genome SNPs (cgSNPs) [31]. The clonal strains differed by fewer than four cgSNPs [12]. Recombinogenic regions were removed with Gubbins v2.3.4 [32]. To construct a maximum likelihood phylogeny of the sequenced isolates, RAxML v8.2.10 was used with the generalized time-reversible model and a GTRGAMMA distribution to model site-specific rate variation [33]. We used iTOL [34] to visualize and edit the phylogenetic tree.

### Data availability

All the 154 whole-genome sequenced data have been deposited in the NCBI database BioProject: PRJNA550041. A total of 34 *bla*_KPC-2_-carrying or virulence plasmid sequences were deposited in the GenBank database and assigned the accession numbers MT269819-MT269852.

## Results

### Kp is the dominant CRE species from BSI patients

A total of nine tertiary hospitals were involved in this study, with a median of 1,851 beds (IQR 1,375-2,851). Of 425 *Kp* isolates from patients with BSIs collected between 2005 and 2019, 72 were CRKP. The CRKP isolates were most frequently susceptible to colistin (71/72, 99%), followed by tigecycline (60/72, 83%), and amikacin (38/72, 53%) (Figure S1A). The carriage of *rmtB* and *armA* contributed to the resistance of amikacin and other aminoglycoside antibiotics, while the resistance of quinolones was mostly attributed to plasmid-mediated *qnrB* and *qnrS1* genes. Furthermore, the MICs were significantly higher among CRKP for multiple antibiotics (Figure S1B; Table S4).

### A widespread ST11-bla_KPC-2_ lineage in Kp

**Figure 1.**
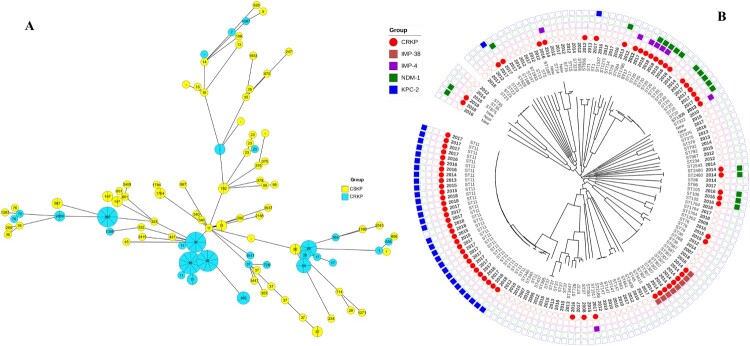
Population structure of CRKP and CSKP isolates. (A) Minimum-spanning tree of *Kp* isolates based on a core-genome MLST (cgMLST). Each node within the tree represents a cgMLST type, with diameters scaled to the number of isolates belonging to that type. Join lines represent locus variants. The length of the branch between each node is proportional to the number of distinct alleles of cgMLST scheme genes that differ between the two linked nodes. The figure was colored by the group of strains and each node showed labels of STs. (B) Phylogeny of core genome SNPs in 147 KpI isolates. The classic virulence ST23-KL1 strain NTUH-K2044 (GenBank accession no. NC_012731) was used as a reference. The circle beside the nodes indicate strains of carbapenem-resistant *Klebsiella pneumoniae* (CRKP) (solid) and carbapenem-susceptible *Klebsiella pneumoniae* (CSKP) (hollow). Squares indicate the carbapenemase-encoding gene to be given beside the relevant phylogeny.

We sequenced 154 *Klebsiella* isolates, including 72 CRKP and 82 CSKP. Among these isolates, there were 62 distinct STs, and seven strains with novel alleles. The most prevalent STs were ST11 (n=34), ST20 (n=13), ST307 (n=8), ST37 (n=7), ST147 (n=4), and ST23 (n=4) (Table S1). Each ST identified in multiple isolates was more common in CRKP strains, while most of the singleton STs were CSKP isolates (Figure 1A). Furthermore, ST11, ST307, ST76, ST37, ST23, ST20, ST1473, ST1, ST656, and ST14 included both CRKP and CSKP isolates. According to cgMLST, ST11 and ST20 strains can be divided into a few sub-types, while all the ST307 strains were clustered into the same cgMLST type. The ST11 *Kp* was assigned in 34 isolates from four hospitals across eight years. Out of these 34 isolates, 31 were CRKP, of which 30 harboured *bla*_KPC-2_ with the remaining strain lacking any known carbapenemase-encoding gene. Identification of capsule synthesis loci revealed that KL47 (21/154, 14%), KL64 (16/154, 10%), KL102 (12/154, 8%), and KL28 (12/154, 8%) were the most common loci. ST11 *Kp* strains included 21 ST11-KL47, nine ST11-KL64, two ST11-KL111, one ST11-KL15, and one unknown capsule type (Table S1).

By maximum likelihood (ML) phylogenetic analysis derived from the core genome SNPs among the 154 *Kp* isolates, four phylogroups of KpI (*Kp*, n = 147), KpII (*Klebsiella quasipneumoniae*, n = 5), KpIII (*Klebsiella quasivariicola*, n = 1), and KpIII (*Klebsiella variicola*, n = 1) were observed. The population structure of the 147 KpI isolates, including 76,408 SNPs extracted from 3,617,098 bp sequences concatenated from 3,780 core genes was explored. Phylogenetic analyses revealed a deep branching and scattered population structure that was broadly classified into distinct phylogenetic lineages (Figure 1B). In contrast, the 76 CSKP isolates were unclustered and intermingled with the 71 CRKP isolates. Notably, all the ST11-CRKP isolates were clustered as the dominant phylogroup with limited nucleotide divergences among isolates belonging to the same capsular types. However, the other three ST11-CSKP isolates were grouped into two sub-lineages that were phylogenetically distal from the ST11-KL47/KL46 group. Two of these three isolates belonged to KL111, and the other isolate belonged to KL15. Upon the enrollment of reference genomes, the ST11-*bla*_KPC-2_ strains from multiple provinces in China were clustered together (Figure S2). Furthermore, the two ST11-KL111 isolates in the collection clustered together with an ST11-KL64 isolate from Singapore, two isolates from Germany, and one isolate from Spain, while the ST11-KL15 strain was clustered together with KL15 strains from Europe [18,19].

### Nosocomial outbreak and transmission caused by ST11 and ST307 CRKP

**Figure 2.**
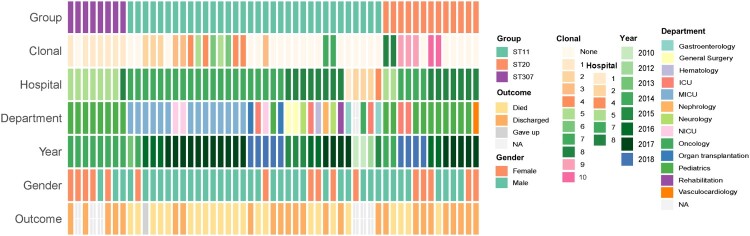
Epidemiological data and clonal identification of ST11, ST307, and ST20 *Kp* isolates. All the ST11 (n = 34), ST20 (n = 13), and ST307 (n = 8) isolates were enrolled and each block represented a strain which was ordered by the sampling date from the same hospital within each group of STs. For the clonal panel, clonal strains were indicated when the difference of core-genome SNPs fewer than four. Each number in the legend represented one type of collections of clonal strains, and the strain marked the same number/color represented that they are clonal-related strains.

Considering the strains within each phylogroup of ST11, ST20, and ST307 differed by a few cgSNPs (Figure S3), their link to a nosocomial outbreak of infection was investigated. Out of the eight neonatal infections caused by ST307 CRKP, seven infants were admitted to the pediatric intensive care unit (PICU) in the same hospital (Figure 2). The duration of the infections lasted from 28 days to 63 days, and the seven ST307 CRKP were isolated within one week of admission to the hospital. (Table S1). Pairwise SNP analysis of the seven ST307 CRKP showed that six of them differed by fewer than 4 cgSNPs, indicating that these strains originated from a single clone. The remaining ST307 strain from this hospital exhibited a difference of 10–12 cgSNPs compared to the other six isolates. The integrated genomic and epidemiological analysis suggested that ST307 CRKP strains were linked to a nosocomial outbreak of infection. The 13 ST20 *Kp* strains were collected between 2014 and 2018 across three different hospitals. The isolates from the same hospital had fewer cgSNPs differences and multiple clones were identified across the isolates (Figure 2). Furthermore, 21 out of the 34 ST11 *Kp* isolates were collected from the same hospital from 2013 to 2018 and the majority of the patients were admitted to the MICU (67%, 14/21) (Figure 2; Table S1). 14 out of the 21 strains were related to multiple clones with cgSNPs ≤ 4. However, since ST11 *Kp* is prevalent in China, and given the limited strain numbers and time lag between patient presentations, both nosocomial transmission events and independent introductions in each hospital were possible.

### Extensive ISKpn26 insertion within the AcrAB-TolC repressor acrR contributes to the multidrug-resistant (MDR) phenotypes in ST11-bla_KPC-2_ Kp

**Figure 3.**
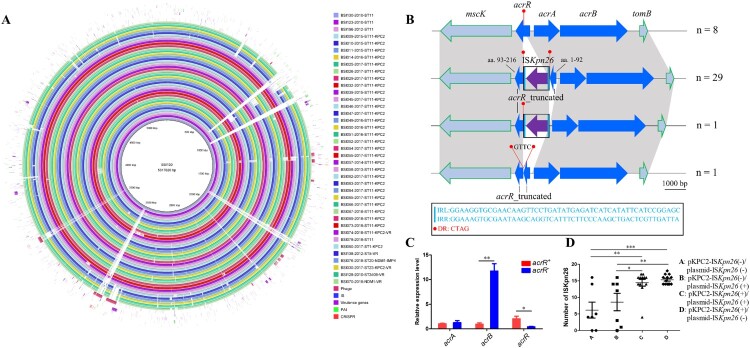
Schematic diagram of mobile genetic elements integrated in ST11-*bla*_KPC-2_ chromosome. (A) Circular genetic map of 39 completely assembled *Kp* genomes. The color intensity in each ring represents the BLASTn match identity to the *Kp* strain BSI130 genome. The distribution of prophage sequences, insertion sequence (IS) elements, virulence genes, pathogenicity island (PAI)-like sequences, and CRISPR sequences were mapped to the BSI130 chromosome. The legend of each ring indicates the strain ID, year of isolation, sequence type (ST), carbapenemase-encoding gene, plasmid-mediated virulence gene (VR). (B) Schematic presentation of IS*Kpn26* insertion into the *acrR* gene in *Kp* genomes. This represented three types of IS*Kpn26* insertion, each identified in different *Kp* isolates. All the 39 newly assembled *Kp* genomes were used to clarify the profile of IS*Kpn26* insertion. Homologous sequences (representing >99% sequence identity) are indicated by light gray shading. Arrows show the direction of transcription of open reading frames (ORFs). (C) The relative mRNA expression of *acrR* and *acrAB*. *acrR*^+^ represents three ST11 *Kp* harbouring intact *acrR* without IS*Kpn26* insertion; *acrR*^-^ represents three ST11 *Kp* carrying truncated *acrR* by IS*Kpn26*. Data represent the mean ± SE. (D) The number of IS*Kpn26* in the 39 newly assembled *Kp* chromosomes. The four groups represent *Kp* strains harbouring IS*Kpn26* which was located in different plasmids. Group A represents no IS*Kpn26* found either in KPC-2 plasmid or other plasmids (n = 7); Group B represents IS*Kpn26* found only in non-*bla*_KPC-2_-carrying plasmids (n = 7); Group C represents IS*Kpn26* found both in KPC-2 plasmid and other plasmids (n = 12); Group D represents IS*Kpn26* found only in KPC-2 plasmid (n = 13). (t-test, *P<0.05; **P<0.01; ***P<0.001).

Comparative analysis of 39 completely assembled *Kp* genomes revealed extensive conservation of gene content between ST11-*bla*_KPC-2_ and ST11 *Kp* without *bla*_KPC-2_ (Figure 3A). However, three additional segments of ∼28-kbp, ∼7,600-bp, and ∼5,500-bp were exclusively observed in the three ST11 isolates that lacked *bla*_KPC-2_ (Table S5). Furthermore, all ST11 isolates carried a 52-kbp intact prophage sequence named PHAGE_Salmon_SEN34, which was absent from 6 non-ST11 genomes.

**Figure 4.**
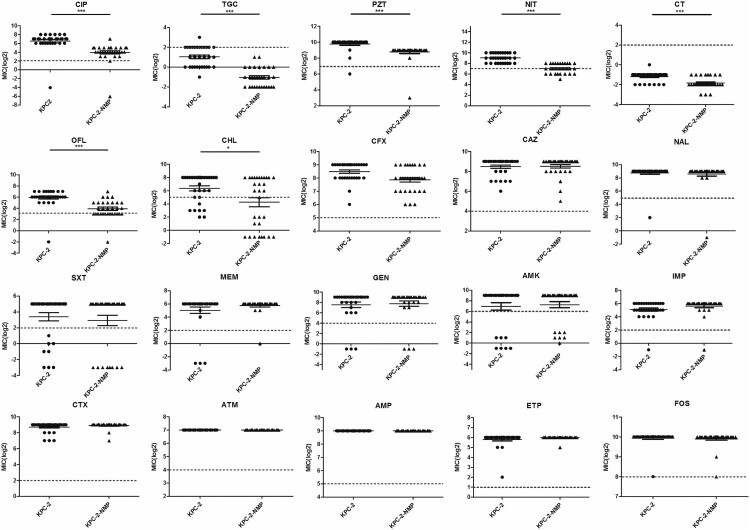
Antimicrobial resistance profile of 20 antimicrobial agents among 30 ST11-*bla*_KPC-2_ *Kp* before and after adding NMP. The MICs were log-transformed for statistical analysis. NMP: 1-(1-naphthylmethyl)-piperazine (NMP). CIP: ciprofloxacin; TGC: tigecycline; PZT: piperacillin-tazobactam; NIT: nitrofurantoin; CT: colistin; OFL: ofloxacin; CHL: chloramphenicol; CFX: cefoxitin; CAZ: ceftazidime; NAL: nalidixic acid; SXT: trimethoprim-sulfamethoxazole; MEM: meropenem; GEN: gentamicin; AMK: amikacin; IMP: imipenem; CTX: cefotaxime; ATM: aztreonam; AMP: Ampicillin; ETP: ertapenem; FOS: fosfomycin. Dashed lines represented resistance breakpoints (t-test, *P<0.05; ***P<0.001).

We further determined the presence of IS elements in the chromosome for those genomes. Notably, we found that 30 out of 39 genomes had an insertion of IS*Kpn26* (1,196 bp, IS*5* family) within the *acrR* gene, all of which were ST11-*bla*_KPC-2_ strains. Among the 30 IS*Kpn26* sequences, ten SNPs were detected and consisted of G+173A, T+176C, A+180G, C+181 T, C+182G, C+184A, T+185A, G+647A, G+698 T, and G+950A. In each strain, the IS*Kpn26* sequence within *acrR* was identical to the IS*Kpn26* located in the *bla*_KPC-2_-carrying plasmid. IS*Kpn26* insertion was located in the target site of a 4-bp (CTAG) direct repeat (DR) at +276 bp of *acrR*, with a target site duplication producing another copy of DR at the boundaries of IS*Kpn26* after its transposition (Figure 3B). Besides the 29 strains with the consistent insertion of IS*Kpn26* forming two Δ*acrR* fragments, one isolate harboured an additional insertion causing the loss of the first 276-bp sequence of *acrR* and the duplicated DR. Although the remaining ST11-*bla*_KPC-2_ isolate lacked IS*Kpn26* within *acrR*, it harboured two copies of DR and a 4-bp (GTTC) sequence belonging to IS*Kpn26*, indicating that IS*Kpn26* had been inserted in *acrR*. By searching all the additional newly assembled *Kp* genomes (n = 117), IS*Kpn26* insertion in *acrR* was not detected. By searching all the 669 fully assembled *Kp* genomes in the public database, the intact IS*Kpn26* insertion in *acrR* was found in 85 isolates. The vast majority of these isolates were collected in China and 80 were ST11 strains (Table S6). qPCR showed that IS*Kpn26* interruption blocked the expression of *acrR* (*P* = 0.032, t-test), while the relative expression of *acrB* was enhanced by a 12.6-fold change (*P *= 0.002, t-test) (Figure 3C). The susceptibility testing among all the ST11-*bla*_KPC-2_ isolates revealed significantly reduced MICs for multiple antimicrobial agents; namely tigecycline, ciprofloxacin, colistin, piperacillin-tazobactam, nitrofurantoin, ofloxacin, and chloramphenicol in the presence of the efflux pump inhibitor NMP (*P *< 0.05) (Figure 4). Next integration of IS*Kpn26* into other regions of the chromosome was assessed. It was found that the strains which harboured plasmids with both IS*Kpn26* and *bla*_KPC-2_, had a significantly higher mean number of IS*Kpn26* in the chromosome (15 ± 0.4 vs. 6 ± 2.4, *P* < 0.001, t-test) (Figure 3D).

### MDR-virulent plasmids and virulence plasmids in bla_KPC-2_-harbouring Kp

**Figure 5.**
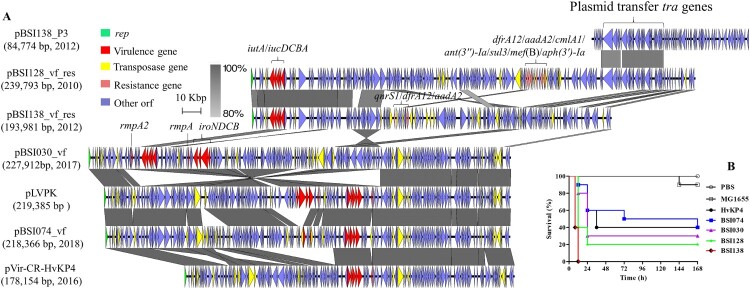
Virulence plasmids and their relevance with pathogenicity. (A) Detailed comparison of linear maps of virulence plasmids in *Kp* isolates. Two classical virulence plasmids of pLVPK (AY378100) and pVir-CR-HvKP4 (MF437313) were used as references. Dark gray shading indicates homologous regions. Arrows show the direction of transcription of open reading frames (ORFs). Genes, mobile elements, and other features are colored based on function classification. The virulence and resistance genes are marked. The figure is drawn to scale. (B) Kaplan-Meier survival curves for seven-day mortality following *Kp* infections. Each group had 10 larvae in the *G. mellonella* infection model. Controls included a PBS injection group, a non-virulent control using *E. coli* MG1655, and a highly-virulent control HvKP4. The survival curve was created using GraphPad Prism.

We further detected the virulence plasmid-harbouring genes of *rmpA* (hypermucoidy) (CRKP=4; CSKP=14), *rmpA2* (hypermucoidy) (CRKP=0; CSKP=1), *iucABCD*/*iutA* (aerobactin) (CRKP=4; CSKP=15), and *iroBCDN* (salmochelin) (CRKP=1; CSKP=14). Of the four CRKP strains harbouring plasmid-mediated virulence genes, two carried *bla*_KPC-2_ and two carried *bla*_NDM-1_. Complete virulence plasmid sequences for the two KPC-2-producing strains and two earlier CSKP strains (Figure 5A) were obtained. Notably, pBSI128_vf_res (239,793 bp) and pBSI138_vf_res (193,981 bp), cultured from patients in 2010 and 2012 at the same hospital, carried both virulence factors (*iucABCD* and *iutA*) and multiple ARGs (Figure 5A). These two plasmids had IncFIB and IncFII replicons and displayed >90% sequence identity with 60% coverage. In the two plasmids, *iuc* and *iutA* were associated with an IS*Ec45* downstream. They shared sequence identities across the virulence module, while significant differences were observed in the MDR region. The 14,922-bp MDR region in pBSI128_vf_res contained genes conferring resistance to trimethoprim, chloramphenicol, aminoglycoside, and macrolides. Furthermore, the pBSI138_P3 from *Kp* strain BSI138 shared two homologous regions with pBSI128_vf_res, which covered the *tra* loci, one 21,497 bp in length with 97% identity and another 9,397 bp in length with 94% identity. A BLASTn search did not find homologous plasmids to pBSI138_vf_res and pBSI128_vf_res (coverage <60%), with the exception of p130411-38618_1 (GenBank: MK649826) from a *Kp* isolate collected in 2011 in Vietnam [35] (identity >99%, coverage 62%).

Among the two KPC-2-producing strains ST23 BSI030 and ST11 BSI074, pBSI030_vf harbours the virulence genes of *iuc*, *iro*, *iutA*, *rmpA* and *rmpA2*. This plasmid backbone showed similarity to the classic virulence plasmid pLVPK (>99% nucleotide identity, 93% coverage) but with multiple inverted regions (Figure 5A). Furthermore, pBSI030_vf carried an 11,716-bp fragment containing HigB/HigA toxin/antitoxin system that was not present in pLVPK. The pBSI074_vf backbone was similar to pBSI030_vf (>99% identity, 97% coverage) and pLVPK (>99% identity, 90% coverage) but lacked *iroBCD* and *rmpA2* genes, and *rmpA* and *iroN* genes were truncated by IS*Kpn26*. Rearrangement of multiple IS elements in pBSI074_vf also resulted in the difference in the recently identified pVir-CR-HvKP4 in China (Figure 5A). Using the *G. mellonella* infection model, we demonstrated that all four strains harbouring virulence plasmids are highly virulent compared with the hypervirulent strain HvKP4 (Figure 5B).

### The highly diverse structure of bla_KPC-2_-carrying plasmids

**Figure 6.**
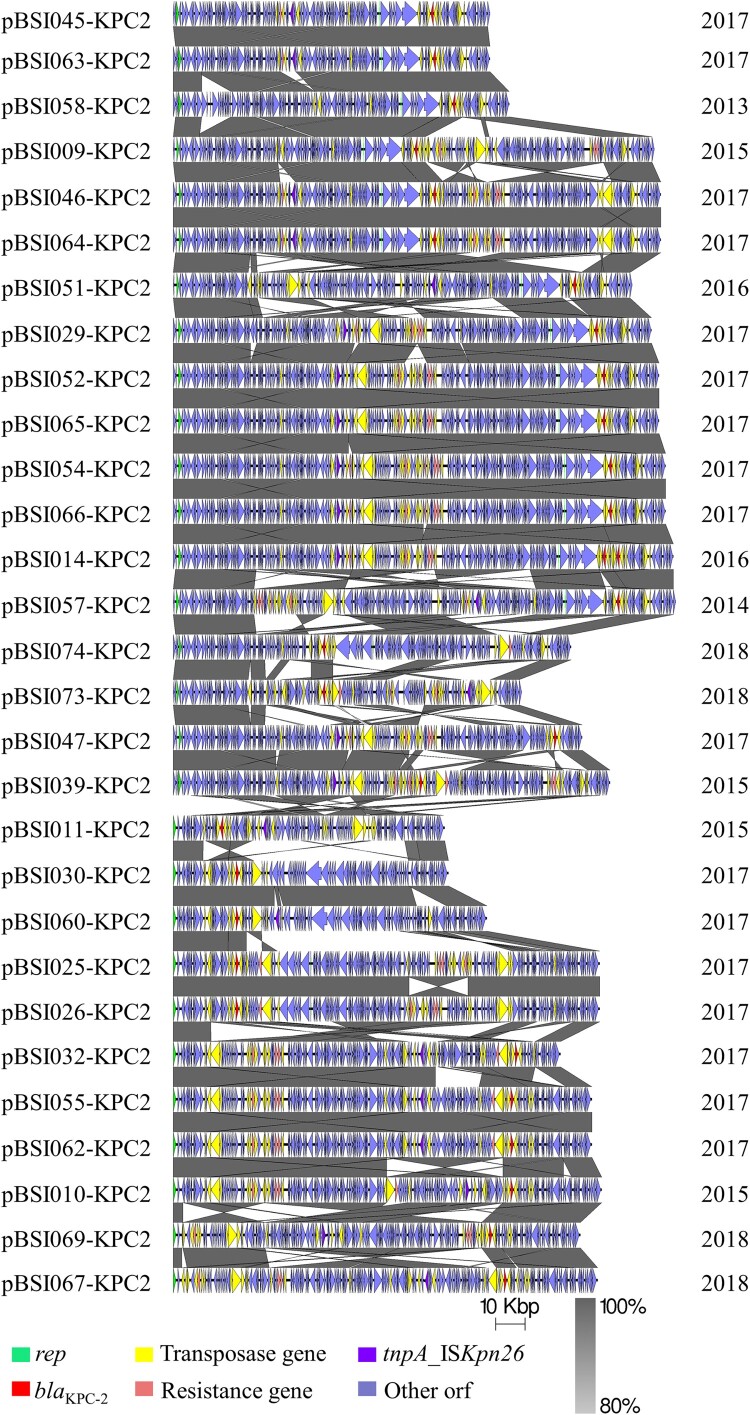
The highly diverse structure of *bla*_KPC-2_-carrying plasmids in *Kp*. The figure represents major structural features of 29 completed *bla*_KPC-2_-carrying plasmids. ORFs are portrayed by arrows to indicate the direction of transcription and colored based on their predicted gene functions. Dark gray shading indicates homologous regions. The name of each plasmid and related year of isolation are highlighted. The figure is drawn to scale.

Among the 72 CRKP isolates, *bla*_KPC-2_ was the predominant type and detected in 32 isolates. S1-PFGE and Southern blotting hybridization indicated that *bla*_KPC-2_ genes were located on plasmids with diverse sizes in the 32 *Kp* isolates (Figure S4). To further clarify the features of *bla*_KPC-2_-carrying plasmids, 29 complete plasmid sequences were obtained from the 32 *bla*_KPC-2_-harbouring *Kp* (ST11 = 30; ST23 = 1; ST1 = 1). The sizes of plasmids ranged from 92,603 bp for pBSI011-KPC2 to 171,483 bp for pBSI057-KPC2 (Figure 6). Almost all (28/29) of the plasmids carried at least one copy of the pilin-coding gene *traA*. The *bla*_KPC-2_ gene was surrounded by a 4,655-bp sequence consisting of IS*26* (820 bp)-*tnpR* (402 bp)-IS*Kpn27* (1,080 bp)-*bla*_KPC-2_ (882 bp)-ΔIS*Kpn6* (1,033 bp) across all the sequenced plasmids. No other resistance genes were detected in five plasmids, while *catA2*, *bla*_TEM-1B_, *rmtB*, *bla*_CTX-M-65_, *fosA3*, and *bla*_SHV-12_ were integrated into the other plasmids (Figure S5). These genes were found in concomitant plasmids within the *bla*_KPC-2_-harbouring isolates, indicating that *bla*_KPC-2_-carrying plasmids are plastic and can capture ARGs from adjacent plasmids in the same host.

## Supplementary Material

Supplemental Material

## Discussion

The *Kp* isolates have evolved separately in distinct clonal groups, including the virulent clonal ST23, and the MDR clonal ST258 and ST11 [36]. It is important to identify carbapenem-resistant hypervirulent *Kp* and to understand the risk of transmission. Among the *Kp* strains from BSI patients, potentially novel, differently sized virulence plasmids associated with high mortality of *G. mellonella* were identified. Two KPC-2-producing strains (ST11 BSI074 and ST23 BSI030) harbouring virulence plasmids were detected, and pBSI074_vf shared high similarity to pBSI030_vf. Considering that a virulence plasmid was rarely found in ST11 strains but was prevalent in ST23 isolates in our collection (100%) and other sources [10], pBSI074_vf was likely acquired from ST23 isolates. Notably, two MDR-virulent plasmids harbouring virulence markers *iucABCDiutA* plus multiple ARGs were identified. These plasmids also possess plasmid transfer-associated genes that probably facilitate their dissemination. MDR-virulent plasmids were rare but an increasing number of studies have reported such strains in the past few years [35,37,38,39]. The emergence and spread of MDR-virulent plasmids as a result of ongoing recombination is a significant potential health threat in terms of both disease and treatment.

ST11 *Kp* is the dominant sequence type in China [12], which was distinct from the prevalence in other countries in South and Southeast Asia [35]. In Europe, ST11 *Kp* was accounting for approximately 10% of the clinical *Kp* population, and *bla*_NDM-1_ and *bla*_OXA-48_ are the dominant genes in ST11 CRKP [18]. In this study, the extensively disseminated ST11-*bla*_KPC-2_ *Kp* was continuously identified in BSIs patients. The nosocomial transmission has been detected in some hospitals. The *bla*_KPC-2_-carrying plasmids are highly diverse, and ongoing integration of additional resistance genes has been identified as causing the transmission of MDR plasmids. Another finding is the extensive integration of IS*Kpn26* into *acrR*, which was observed in all the ST11-*bla*_KPC-2_ genomes, leading to the deactivation of *acrR* and increased expression of *acrB*. IS*Kpn26*-like insertion in *mgrB* has been found in ST258 *Kp* which was responsible for colistin resistance [40]. IS*Kpn26* insertion in *acrR* has only been reported in an ST11-*bla*_KPC-2_ *Kp* in Taiwan [41]. We found that the intact IS*Kpn26* insertion in *acrR* mostly happened in strains from Asia. Furthermore, a replicative-like transposition [42] produced 14–18 copies of IS*Kpn26* in the chromosome. A strong link between KPC-2 plasmid-located IS*Kpn26* and IS*Kpn26* insertion into *acrR* indicates that *bla*_KPC-2_ plasmid is the reservoir for IS*Kpn26*. The *acrR* gene is the local repressor of resistance-nodulation-division (RND) efflux pump AcrAB-TolC, which is critical to acquire antimicrobial resistance (AMR) [43,44], and is involved in virulence [45]. We found that the presence of NMP increased the susceptibility of multiple antibiotics, including the last-resort antibiotic tigecycline to treat CRE infections. These data demonstrated that IS*Kpn26* insertion contributed to the MDR phenotypes in ST11-*bla*_KPC-2_ *Kp* by blocking the expression of AcrAB-TolC repressor *acrR*. The interaction between the *bla*_KPC-2_ plasmid and the ST11 *Kp* host may, therefore, facilitate nosocomial dissemination and transfer of AMR.

In conclusion, our results revealed a widespread ST11-*bla*_KPC-2_ lineage of CRKP from BSI patients in China. In addition to contributing to AMR spread, KPC-2 plasmids can further interact with the host and alter some host-dependent social traits, such as the provision of IS*Kpn26* to insert into *acrR*, and therefore, up-regulate AcrAB-TolC multidrug efflux pump, leading to the increased MDR phenotypes of the host bacterium. Furthermore, we identified novel MDR-virulent plasmids due to ongoing recombination in *Kp*; therefore, representing a significant health threat in terms of both disease and treatment.
