# Supplementary material for: Molecular characterization of carbapenem-resistant and virulent plasmids in Klebsiella pneumoniae from patients with bloodstream infections in China
Source: Emerg Microbes Infect. 2021 Apr 5;10(1):700–9. doi: 10.1080/22221751.2021.1906163 (PMC8023600; doi:10.1080/22221751.2021.1906163)
Supplement: Supplemental Material [file TEMI_A_1906163_SM8894.zip › Supplemental files/Table S2.docx]

**Table S2 The primers used in qPCR**

| Primers | Sequence (5’-3’) | Amplicon size (bp) | Reference |
| --- | --- | --- | --- |
| *acrA* | GCCGGAAGTCGGTATTGTGA | 274 | In this study |
|  | ACGCTTGACCGTCAGTTGAT |  |  |
| *acrB* | TGTCGGCGTCATTAACACCA | 208 | In this study |
|  | TTTCAGGGCGCTGATCACAT |  |  |
| *acrR* | CTTGAAAGAGTGCATCGCGG | 135 | In this study |
|  | GCATGCAGGTCGAACGAATC |  |  |
| 16S rRNA | CGGTGAATACGTTCYCGG | 143 | [1] |
|  | GGWTACCTTGTTACGACTT |  |  |

Reference:

1. Gaze, W.H. *et al.* Impacts of anthropogenic activity on the ecology of class 1 integrons and integron-associated genes in the environment. *ISME J.* **5**, 1253-1261 (2011).
