# Supplementary material for: Molecular characterization of carbapenem-resistant and virulent plasmids in Klebsiella pneumoniae from patients with bloodstream infections in China
Source: Emerg Microbes Infect. 2021 Apr 5;10(1):700–9. doi: 10.1080/22221751.2021.1906163 (PMC8023600; doi:10.1080/22221751.2021.1906163)
Supplement: Supplemental Material [file TEMI_A_1906163_SM8894.zip › Supplemental files/Table S3.docx]

**Table S3.** The information of 162 references *K. pneumoniae* KpI isolates

| **Accession no.** | **Strain ID** | **Strain_name** | **Fixed_strain_name** | **Phylogroup** | **ST** | **K locus** | **Country_isolated** | **Year_isolated** | **Source_Host** | **Sample_note** |
| --- | --- | --- | --- | --- | --- | --- | --- | --- | --- | --- |
| ERS011807 | SKP000322 |  | H137 | KpI | 11 | KL104 | Vietnam | 2007 | Human | bronchial alveolar lavage |
| ERS011902 | SKP000724 |  | H78 | KpI | 11 | KL64 | Singapore | 2004 | Human | foot |
| ERS011904 | SKP000726 |  | H80 | KpI | 11 | KL105 | Singapore | 2004 | Human | urine |
| ERS011906 | SKP000729 |  | H82 | KpI | 11 | KL38 | Singapore | 2005 | Human | gastric aspirate |
| ERS011907 | SKP000730 |  | H83 | KpI | 11 | KL105 | Singapore | 2004 | Human | urine |
| ERS011911 | SKP000734 |  | H85 | KpI | 11 | KL105 | Singapore | 2005 | Human | urine |
| AKAC01 | KPNIH10 | KPNIH10 | KPNIH10 | KpI | 258 | KL107 | USA | 2011 | Human | groin |
| AJZV01 | KPNIH2 | KPNIH2 | KPNIH2 | KpI | 258 | KL107 | USA | 2011 | Human | wound |
| AKAK01 | KPNIH20 | KPNIH20 | KPNIH20 | KpI | 258 | KL107 | USA | 2011 | Human | throat |
| AKAM01 | KPNIH22 | KPNIH22 | KPNIH22 | KpI | 258 | KL107 | USA | 2011 | Human | rectal swab |
| AJZY01 | KPNIH6 | KPNIH6 | KPNIH6 | KpI | 258 | KL107 | USA | 2011 | Human | peritoneal fluid |
| AJZZ01 | KPNIH7 | KPNIH7 | KPNIH7 | KpI | 258 | KL107 | USA | 2011 | Environmental | ventilator |
| SAMN07259325 |  | 5 | SAMN07259325 | KpI | 11 | KL47 | China | 2016 | Human |  |
| SAMN07259326 |  | 4 | SAMN07259326 | KpI | 11 | KL47 | China | 2016 | Human |  |
| SAMN07259327 |  | 2 | SAMN07259327 | KpI | 11 | KL47 | China | 2016 | Human |  |
| SAMN07259328 |  | 1 | SAMN07259328 | KpI | 11 | KL47 | China | 2016 | Human |  |
| SAMN07259329 |  | 3 | SAMN07259329 | KpI | 11 | KL47 | China | 2016 | Human |  |
| SAMN07259330 |  | FJ8 | SAMN07259330 | KpI | 11 | KL47 | China |  | Human |  |
| SAMN07259331 |  | FJ9 | SAMN07259331 | KpI | 11 | KL47 | China |  | Human |  |
| SAMN07259332 |  | 1088 | SAMN07259332 | KpI | 23 | KL1 | China | 2013 | Human |  |
| SAMN07259333 |  | SH1 | SAMN07259333 | KpI | 11 | KL47 | China | 2015 | Human |  |
| NC_012731 |  | NTUH-K2044 | NTUH-K2044 | KpI | 23 | KL1 | China_Taiwan |  | Human | liver abscess and meningitis |
| ERR1204814 | EuSCAPE_AT002 | 11847 |  | KpI | 11 | KL15 | Austria | 2014 | Human | Wound Secretion |
| ERR1204827 | EuSCAPE_AT023 | A2516 |  | KpI | 11 | KL15 | Austria | 2014 | Human | Lower Respiratory Tract Secrection |
| ERR1204830 | EuSCAPE_AT032 | V15669 |  | KpI | 11 | KL15 | Austria | 2013 | Human | Puncture Fluids |
| ERR1204832 | EuSCAPE_EE005 | 1170 |  | KpI | 11 | KL105 | Estonia | 2013 | Human | Urine |
| ERR1204815 | EuSCAPE_AT004 | 202556 |  | KpI | 11 | KL15 | Austria | 2014 | Human | Urine |
| ERR1204842 | EuSCAPE_PL002 | 1540/14 |  | KpI | 11 | KL105 | Poland | 2014 | Human | Lower Respiratory Tract Secrection |
| ERR1204845 | EuSCAPE_PL007 | 1545/14 |  | KpI | 11 | KL105 | Poland | 2014 | Human | Urine |
| ERR1204846 | EuSCAPE_PL008 | 207/14 |  | KpI | 11 | KL105 | Poland | 2013 | Human | Urine |
| ERR1204847 | EuSCAPE_PL010 | 209/14 |  | KpI | 11 | KL105 | Poland | 2014 | Human | Urine |
| ERR1204852 | EuSCAPE_PL028 | 5786/13 |  | KpI | 11 | KL105 | Poland | 2013 | Human | Urine |
| ERR1204817 | EuSCAPE_AT006 | 2772 |  | KpI | 11 | KL15 | Austria | 2014 | Human | Wound Secretion |
| ERR1204854 | EuSCAPE_PL030 | 622/14 |  | KpI | 11 | KL105 | Poland | 2014 | Human | Blood |
| ERR1204855 | EuSCAPE_PL031 | 701/14 |  | KpI | 11 | KL105 | Poland | 2013 | Human | Puncture Fluids |
| ERR1204856 | EuSCAPE_PL032 | 702/14 |  | KpI | 11 | KL105 | Poland | 2014 | Human | Lower Respiratory Tract Secrection |
| ERR1204857 | EuSCAPE_PL019 | 2895/14 |  | KpI | 11 | KL105 | Poland | 2014 | Human | Urine |
| ERR1204858 | EuSCAPE_PL021 | 2897/14 |  | KpI | 11 | KL105 | Poland | 2014 | Human | Wound Secretion |
| ERR1204859 | EuSCAPE_PL025 | 476/14 |  | KpI | 11 | KL105 | Poland | 2014 | Human | Lower Respiratory Tract Secrection |
| ERR1204862 | EuSCAPE_PL036 | 707/14 |  | KpI | 11 | KL105 | Poland | 2014 | Human | Lower Respiratory Tract Secrection |
| ERR1204863 | EuSCAPE_PL037 | 708/14 |  | KpI | 11 | KL105 | Poland | 2013 | Human | Urine |
| ERR1204865 | EuSCAPE_PL045 | 716/14 |  | KpI | 11 | KL105 | Poland | 2013 | Human | Lower Respiratory Tract Secrection |
| ERR1204867 | EuSCAPE_PL050 | 763/14 |  | KpI | 11 | KL105 | Poland | 2014 | Human | Blood |
| ERR1204868 | EuSCAPE_PL052 | 765/14 |  | KpI | 11 | KL105 | Poland | 2014 | Human | Wound Secretion |
| ERR1204869 | EuSCAPE_PL054 | 781/14 |  | KpI | 11 | KL105 | Poland | 2014 | Human | Lower Respiratory Tract Secrection |
| ERR1204871 | EuSCAPE_PL057 | 786/14 |  | KpI | 11 | KL105 | Poland | 2014 | Human | Urine |
| ERR1204873 | EuSCAPE_PL060 | 840/14 |  | KpI | 11 | KL105 | Poland | 2014 | Human | Urine |
| ERR1204876 | EuSCAPE_FR008 | 13166206 |  | KpI | 11 | KL105 | France | 2013 | Human | Urine |
| ERR1204905 | EuSCAPE_SK001 | 1031 12b |  | KpI | 11 | KL105 | Slovakia | 2014 | Human | Lower Respiratory Tract Secrection |
| ERR1204906 | EuSCAPE_SK002 | 1034 12a |  | KpI | 11 | KL105 | Slovakia | 2014 | Human | Lower Respiratory Tract Secrection |
| ERR1204907 | EuSCAPE_SK003 | 11880 15b |  | KpI | 11 | KL2 | Slovakia | 2014 | Human | Urine |
| ERR1204908 | EuSCAPE_SK004 | 1281 19a |  | KpI | 11 | KL105 | Slovakia | 2014 | Human | Urine |
| ERR1204910 | EuSCAPE_SK006 | 13924/JIS 4a |  | KpI | 11 | KL105 | Slovakia | 2013 | Human | Unknown |
| ERR1204912 | EuSCAPE_SK009 | 14095 JIS 6b |  | KpI | 11 | KL105 | Slovakia | 2013 | Human | Urine |
| ERR1204913 | EuSCAPE_SK010 | 1470 20a |  | KpI | 11 | KL105 | Slovakia | 2014 | Human | Other (tonsil swab) |
| ERR1204914 | EuSCAPE_SK011 | 14750 JIS 7a |  | KpI | 11 | KL24 | Slovakia | 2014 | Human | Urine |
| ERR1204917 | EuSCAPE_SK016 | 16650/2013 2b |  | KpI | 11 | KL105 | Slovakia | 2013 | Human | Lower Respiratory Tract Secrection |
| ERR1204920 | EuSCAPE_SK022 | 19652 21b |  | KpI | 11 | KL105 | Slovakia | 2014 | Human | Urine |
| ERR1204922 | EuSCAPE_SK024 | 2095 11a |  | KpI | 11 | KL2 | Slovakia | 2014 | Human | Urine |
| ERR1204923 | EuSCAPE_SK025 | 2274 11b |  | KpI | 11 | KL2 | Slovakia | 2014 | Human | Urine |
| ERR1204924 | EuSCAPE_SK027 | 2299 23a |  | KpI | 11 | KL105 | Slovakia | 2014 | Human | Urine |
| ERR1204927 | EuSCAPE_SK030 | 2574 24a |  | KpI | 11 | KL105 | Slovakia | 2014 | Human | Urine |
| ERR1204929 | EuSCAPE_SK033 | 3178 16a |  | KpI | 11 | KL2 | Slovakia | 2014 | Human | Wound Secretion |
| ERR1204930 | EuSCAPE_SK034 | 3679 15a |  | KpI | 11 | KL105 | Slovakia | 2014 | Human | Lower Respiratory Tract Secrection |
| ERR1204936 | EuSCAPE_SK044 | 65369/2013 3a |  | KpI | 11 | KL105 | Slovakia | 2013 | Human | Urine |
| ERR1204938 | EuSCAPE_SK046 | 6945 18a |  | KpI | 11 | KL2 | Slovakia | 2014 | Human | Wound Secretion |
| ERR1204940 | EuSCAPE_SK048 | 8284 5b |  | KpI | 11 | KL24 | Slovakia | 2013 | Human | Urine |
| ERR1204943 | EuSCAPE_CZ007 | 3999 |  | KpI | 11 | KL15 | Czech Republic | 2014 | Human | Wound Secretion |
| ERR1204948 | EuSCAPE_CZ013 | B228946 |  | KpI | 11 | KL24 | Czech Republic | 2013 | Human | Urine |
| ERR1204949 | EuSCAPE_CZ014 | B229095 |  | KpI | 11 | KL24 | Czech Republic | 2013 | Human | Urine |
| ERR1204901 | EuSCAPE_FR071 | 2798 |  | KpI | 11 | KL125 | France | 2014 | Human | Urine |
| ERR1204957 | EuSCAPE_CZ025 | D2470 |  | KpI | 11 | KL2 | Czech Republic | 2014 | Human | Lower Respiratory Tract Secrection |
| ERR1204960 | EuSCAPE_CZ032 | D399597 |  | KpI | 11 | KL15 | Czech Republic | 2013 | Human | Urine |
| ERR1204962 | EuSCAPE_CZ034 | F97513 |  | KpI | 11 | KL15 | Czech Republic | 2014 | Human | Urine |
| ERR1204970 | EuSCAPE_AT011 | 351211 |  | KpI | 11 | KL15 | Austria | 2014 | Human | Lower Respiratory Tract Secrection |
| ERR1204984 | EuSCAPE_SK031 | 2806 22a |  | KpI | 11 | KL105 | Slovakia | 2014 | Human | Other (bile) |
| ERR1205013 | EuSCAPE_PL012 | 211/14 |  | KpI | 11 | KL105 | Poland | 2013 | Human | Urine |
| ERR1205015 | EuSCAPE_PL016 | 2519/14 |  | KpI | 11 | KL105 | Poland | 2014 | Human | Urine |
| ERR1205023 | EuSCAPE_PL038 | 709/14 |  | KpI | 11 | KL105 | Poland | 2013 | Human | Lower Respiratory Tract Secrection |
| ERR1205024 | EuSCAPE_PL040 | 711/14 |  | KpI | 11 | KL105 | Poland | 2014 | Human | Urine |
| ERR1205028 | EuSCAPE_PL048 | 761/14 |  | KpI | 11 | KL24 | Poland | 2013 | Human | Urine |
| ERR1204999 | EuSCAPE_EE002 | 1160/2 |  | KpI | 11 | KL105 | Estonia | 2013 | Human | Urine |
| ERR1205001 | EuSCAPE_EE006 | 1213/1 |  | KpI | 11 | KL105 | Estonia | 2013 | Human | Wound Secretion |
| ERR1205067 | EuSCAPE_FR062 | 22865 |  | KpI | 11 | KL125 | France | 2014 | Human | Blood |
| ERR1217059 | EuSCAPE_MT002 | 111914 |  | KpI | 11 | KL15 | Malta | 2013 | Human | Wound Secretion |
| ERR1217066 | EuSCAPE_MT009 | 115563 |  | KpI | 11 | KL15 | Malta | 2013 | Human | Urine |
| ERR1217068 | EuSCAPE_MT011 | 115782 |  | KpI | 11 | KL15 | Malta | 2013 | Human | Urine |
| ERR1217073 | EuSCAPE_MT016 | 119600 |  | KpI | 11 | KL15 | Malta | 2013 | Human | Urine |
| ERR1217074 | EuSCAPE_MT017 | 504715 |  | KpI | 11 | KL15 | Malta | 2013 | Human | Urine |
| ERR1216965 | EuSCAPE_DE019 | NRZ-11715 |  | KpI | 11 | KL15 | Germany | 2014 | Human | Lower Respiratory Tract Secrection |
| ERR1216982 | EuSCAPE_DE036 | NRZ-12547 |  | KpI | 11 | KL105 | Germany | 2014 | Human | Wound Secretion |
| ERR1217005 | EuSCAPE_DE059 | NRZ-14921 |  | KpI | 11 | KL24 | Germany | 2013 | Human | Wound Secretion |
| ERR1217013 | EuSCAPE_DE067 | NRZ-14949 |  | KpI | 11 | KL111 | Germany | 2013 | Human | Wound Secretion |
| ERR1217015 | EuSCAPE_DE069 | NRZ-14951 |  | KpI | 11 | KL111 | Germany | 2012 | Human | Urine |
| ERR1217244 | EuSCAPE_IT058 | 030R(11022590) |  | KpI | 11 | KL24 | Italy | 2013 | Human | Blood |
| ERR1217305 | EuSCAPE_IT127 | 064S(2847423280) |  | KpI | 11 | KL105 | Italy | 2013 | Human | Blood |
| ERR1217330 | EuSCAPE_IT155 | 078S (34790078) |  | KpI | 11 | KL105 | Italy | 2014 | Human | Lower Respiratory Tract Secrection |
| ERR1217478 | EuSCAPE_LV002 | K-1788-13 |  | KpI | 11 | KL105 | Latvia | 2013 | Human | Urine |
| ERR1228227 | EuSCAPE_IT365 | 185S |  | KpI | 11 | KL52 | Italy | 2013 | Human | Urine |
| ERR1228344 | EuSCAPE_ES046 | K2558 |  | KpI | 11 | KL24 | Spain | 2013 | Human | Lower Respiratory Tract Secrection |
| ERR1228345 | EuSCAPE_ES047 | K2559 |  | KpI | 11 | KL24 | Spain | 2013 | Human | Puncture Fluids |
| ERR1228359 | EuSCAPE_ES061 | K2647 |  | KpI | 11 | KL24 | Spain | 2013 | Human | Wound Secretion |
| ERR1228365 | EuSCAPE_ES067 | K2689 |  | KpI | 11 | KL64 | Spain | 2013 | Human | Wound Secretion |
| ERR1228366 | EuSCAPE_ES068 | K2691 |  | KpI | 11 | KL24 | Spain | 2013 | Human | Blood |
| ERR1228371 | EuSCAPE_ES073 | K2707 |  | KpI | 11 | KL13 | Spain | 2013 | Human | Blood |
| ERR1228373 | EuSCAPE_ES075 | K2710 |  | KpI | 11 | KL24 | Spain | 2013 | Human | Puncture Fluids |
| ERR1228380 | EuSCAPE_ES082 | K2753 |  | KpI | 11 | KL24 | Spain | 2013 | Human | Urine |
| ERR1228382 | EuSCAPE_ES084 | K2778 |  | KpI | 11 | KL24 | Spain | 2013 | Human | Wound Secretion |
| ERR1228383 | EuSCAPE_ES085 | K2783 |  | KpI | 11 | KL13 | Spain | 2013 | Human | Blood |
| ERR1228387 | EuSCAPE_ES089 | K2791 |  | KpI | 11 | KL24 | Spain | 2013 | Human | Blood |
| ERR1228391 | EuSCAPE_ES093 | K2802 |  | KpI | 11 | KL105 | Spain | 2014 | Human | Urine |
| ERR1228398 | EuSCAPE_ES100 | K2817 |  | KpI | 11 | KL13 | Spain | 2014 | Human | Puncture Fluids |
| ERR1228264 | EuSCAPE_HU017 | 13-ESC-019 |  | KpI | 11 | KL105 | Hungary | 2013 | Human | Other (glans) |
| ERR1228269 | EuSCAPE_HU022 | 13-ESC-024 |  | KpI | 11 | KL105 | Hungary | 2013 | Human | Wound Secretion |
| ERR1228271 | EuSCAPE_HU024 | 13-ESC-027 |  | KpI | 11 | KL105 | Hungary | 2013 | Human | Blood |
| ERR1228274 | EuSCAPE_HU027 | 13-ESC-038 |  | KpI | 11 | KL105 | Hungary | 2014 | Human | Blood |
| ERR1228278 | EuSCAPE_HU031 | 13-ESC-042 |  | KpI | 11 | KL105 | Hungary | 2014 | Human | Wound Secretion |
| ERR1228280 | EuSCAPE_HU033 | 13-ESC-044 |  | KpI | 11 | KL105 | Hungary | 2014 | Human | Urine |
| ERR1228245 | EuSCAPE_BU022 | 240 |  | KpI | 11 | KL24 | Bulgaria | 2014 | Human | Urine |
| ERR1228298 | EuSCAPE_HU052 | 13-ESC-221 |  | KpI | 11 | KL105 | Hungary | 2013 | Human | Urine |
| ERR1289713 | EuSCAPE_ES279 | PH7 |  | KpI | 11 | KL24 | Spain | 2013 | Human | Urine |
| ERR1289741 | EuSCAPE_GR016 | 5982 |  | KpI | 11 | KL24 | Greece | 2013 | Human | Urine |
| ERR1289747 | EuSCAPE_GR026 | 6013 |  | KpI | 11 | KL24 | Greece | 2014 | Human | Other (central venous catheter) |
| ERR1289748 | EuSCAPE_GR027 | 6016 |  | KpI | 11 | KL24 | Greece | 2014 | Human | Urine |
| ERR1289752 | EuSCAPE_GR031 | 6020 |  | KpI | 11 | KL15 | Greece | 2013 | Human | Urine |
| ERR1289774 | EuSCAPE_GR074 | 6112 |  | KpI | 11 | KL24 | Greece | 2014 | Human | Urine |
| ERR1334560 | EuSCAPE_ES158 | K3078 |  | KpI | 11 | KL10 | Spain | 2014 | Human | Urine |
| ERR1334418 | EuSCAPE_GR001 | 5856 |  | KpI | 11 | KL24 | Greece | 2013 | Human | Blood |
| ERR1334485 | EuSCAPE_GR135 | 6223 |  | KpI | 11 | KL24 | Greece | 2014 | Human | Urine |
| ERR1334424 | EuSCAPE_GR008 | 5934 |  | KpI | 11 | KL24 | Greece | 2013 | Human | Urine |
| ERR1334425 | EuSCAPE_GR010 | 5938 |  | KpI | 11 | KL24 | Greece | 2013 | Human | Urine |
| ERR1334700 | EuSCAPE_PT059 | 20661 |  | KpI | 11 | KL13 | Portugal | 2014 | Human | Urine |
| ERR1334737 | EuSCAPE_PT099 | 20656 |  | KpI | 11 | KL13 | Portugal | 2014 | Human | Unknown |
| ERR1334600 | EuSCAPE_GR121 | 6199 |  | KpI | 11 | KL24 | Greece | 2014 | Human | Urine |
| ERR1334605 | EuSCAPE_GR133 | 6220 |  | KpI | 11 | KL24 | Greece | 2014 | Human | Urine |
| ERR1334606 | EuSCAPE_GR134 | 6222 |  | KpI | 11 | KL24 | Greece | 2014 | Human | Urine |
| ERR1334587 | EuSCAPE_GR094 | 6168 |  | KpI | 11 | KL24 | Greece | 2014 | Human | Wound Secretion |
| ERR1334671 | EuSCAPE_PT025 | 19835 |  | KpI | 11 | KL105 | Portugal | 2014 | Human | Urine |
| ERR1334741 | EuSCAPE_SK013 | 1540 14b |  | KpI | 11 | KL105 | Slovakia | 2014 | Human | Lower Respiratory Tract Secrection |
| ERR1374988 | EuSCAPE_SI014 | EUSCAPE-21 |  | KpI | 11 | KL155 | Slovenia | 2014 | Human | Urine |
| ERR1374875 | EuSCAPE_UK075 | H142380403 |  | KpI | 11 | KL15 | United Kingdom (England, Wales & N. Ireland) | 2014 | Human | Urine |
| ERR1415466 | EuSCAPE_RO007 | 1294 |  | KpI | 11 | KL15 | Romania | 2014 | Human | Blood |
| ERR1415585 | EuSCAPE_RO046 | 61752 |  | KpI | 11 | KL105 | Romania | 2013 | Human | Urine |
| ERR1415592 | EuSCAPE_RO054 | 790/323476 |  | KpI | 11 | KL105 | Romania | 2014 | Human | Lower Respiratory Tract Secrection |
| ERR1415611 | EuSCAPE_RO073 | AO5900 |  | KpI | 11 | KL105 | Romania | 2014 | Human | Urine |
| ERR1415617 | EuSCAPE_RO079 | AQ0721 |  | KpI | 11 | KL110 | Romania | 2014 | Human | Urine |
| ERR1415549 | EuSCAPE_UK041 | H140940769 |  | KpI | 11 | KL24 | United Kingdom (England, Wales & N. Ireland) | 2014 | Human | Urine |
| ERR1415671 | EuSCAPE_HR013 | 13280 |  | KpI | 11 | KL105 | Croatia | 2014 | Human | Blood |
| ERR1514961 | EuSCAPE_IE019 | ME140082 |  | KpI | 11 | KL15 | Ireland | 2014 | Human | Urine |
| ERR1514921 | EuSCAPE_BE100 | CNR20140270 |  | KpI | 11 | KL105 | Belgium | 2014 | Human | Lower Respiratory Tract Secrection |
| ERR1541394 | EuSCAPE_TR141 | H2.1R |  | KpI | 11 | KL125 | Turkey | 2013 | Human | Blood |
| ERR1541401 | EuSCAPE_TR148 | H4R |  | KpI | 11 | KL125 | Turkey | 2013 | Human | Puncture Fluids |
| ERR1541266 | EuSCAPE_BE111 | CNR20140430 |  | KpI | 11 | KL105 | Belgium | 2014 | Human | Urine |
| ERR1541544 | EuSCAPE_PL004 | 1542/14 |  | KpI | 11 | KL105 | Poland | 2014 | Human | Urine |
| ERR1541548 | EuSCAPE_FR049 | 17959 |  | KpI | 11 | KL105 | France | 2014 | Human | Blood |
| ERR1541549 | EuSCAPE_SK008 | 14091 JI 6a |  | KpI | 11 | KL105 | Slovakia | 2013 | Human | Urine |
| ERR1541550 | EuSCAPE_SK012 | 14831 7b |  | KpI | 11 | KL105 | Slovakia | 2014 | Human | Wound Secretion |
| ERR1541551 | EuSCAPE_SK018 | 1703 9a |  | KpI | 11 | KL105 | Slovakia | 2014 | Human | Wound Secretion |
| ERR1541553 | EuSCAPE_SK026 | 2287 10a |  | KpI | 11 | KL2 | Slovakia | 2014 | Human | Urine |
| ERR1541557 | EuSCAPE_CZ016 | BUR-30415 |  | KpI | 11 | KL24 | Czech Republic | 2013 | Human | Urine |
| ERR1541559 | EuSCAPE_CZ026 | D2732 |  | KpI | 11 | KL2 | Czech Republic | 2014 | Human | Lower Respiratory Tract Secrection |
| ERR1541432 | EuSCAPE_TR184 | KR4 |  | KpI | 11 | KL15 | Turkey | 2013 | Human | Urine |
