# Supplementary figures and images for: Molecular characterization of carbapenem-resistant and virulent plasmids in Klebsiella pneumoniae from patients with bloodstream infections in China
Source: Emerg Microbes Infect. 2021 Apr 5;10(1):700–9. doi: 10.1080/22221751.2021.1906163 (PMC8023600; doi:10.1080/22221751.2021.1906163)

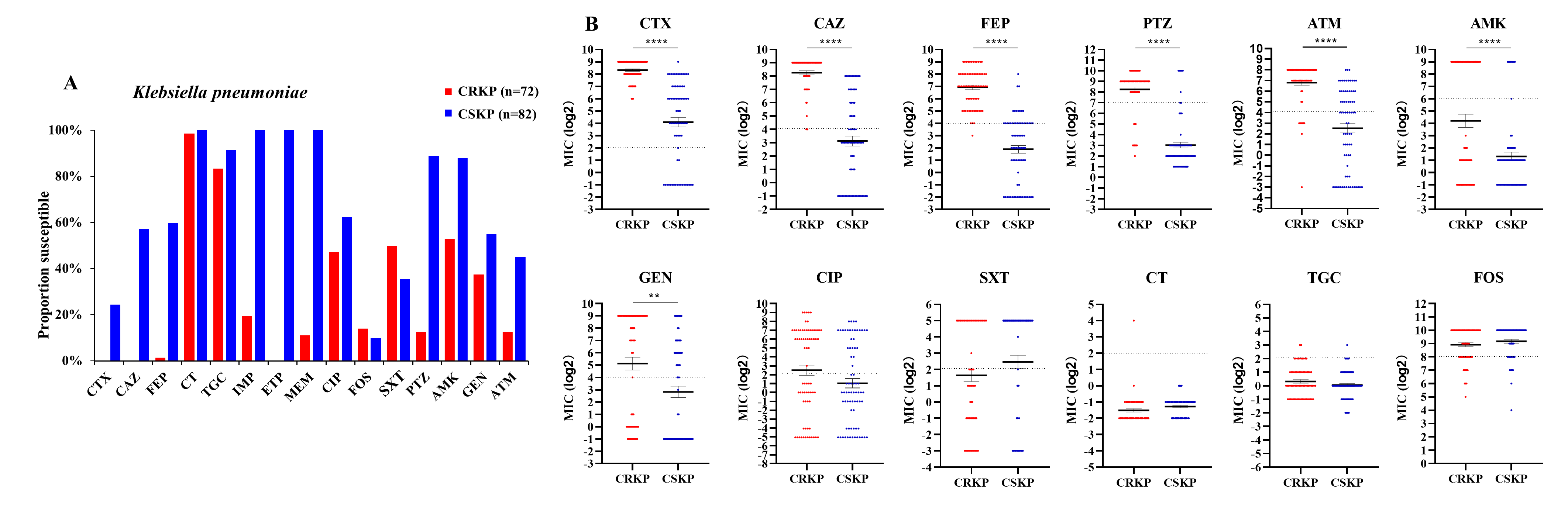

Supplement: Supplemental Material [file TEMI_A_1906163_SM8894.zip › Supplemental files/Figure S1.tif]

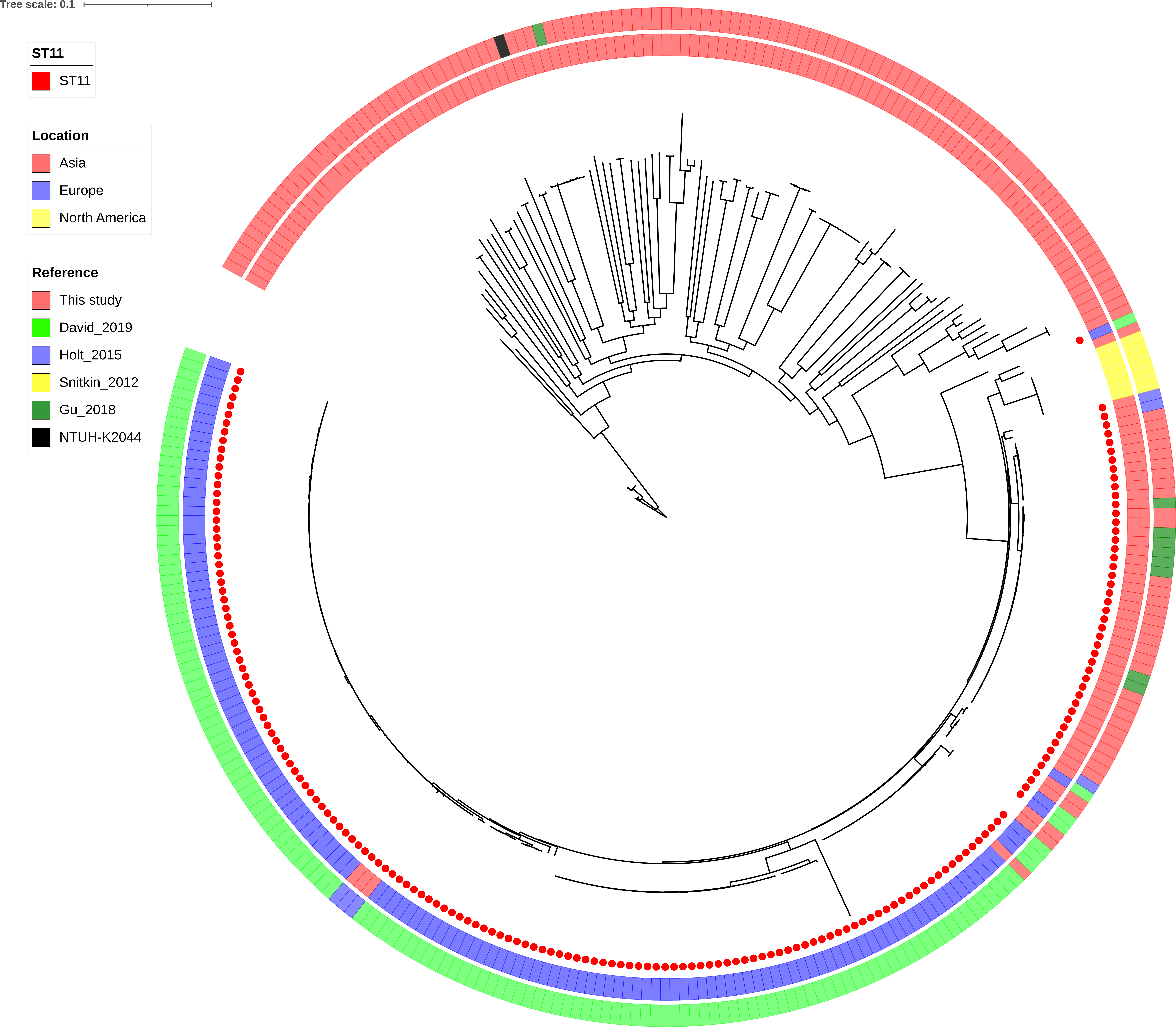

Supplement: Supplemental Material [file TEMI_A_1906163_SM8894.zip › Supplemental files/Figure S2.tif]

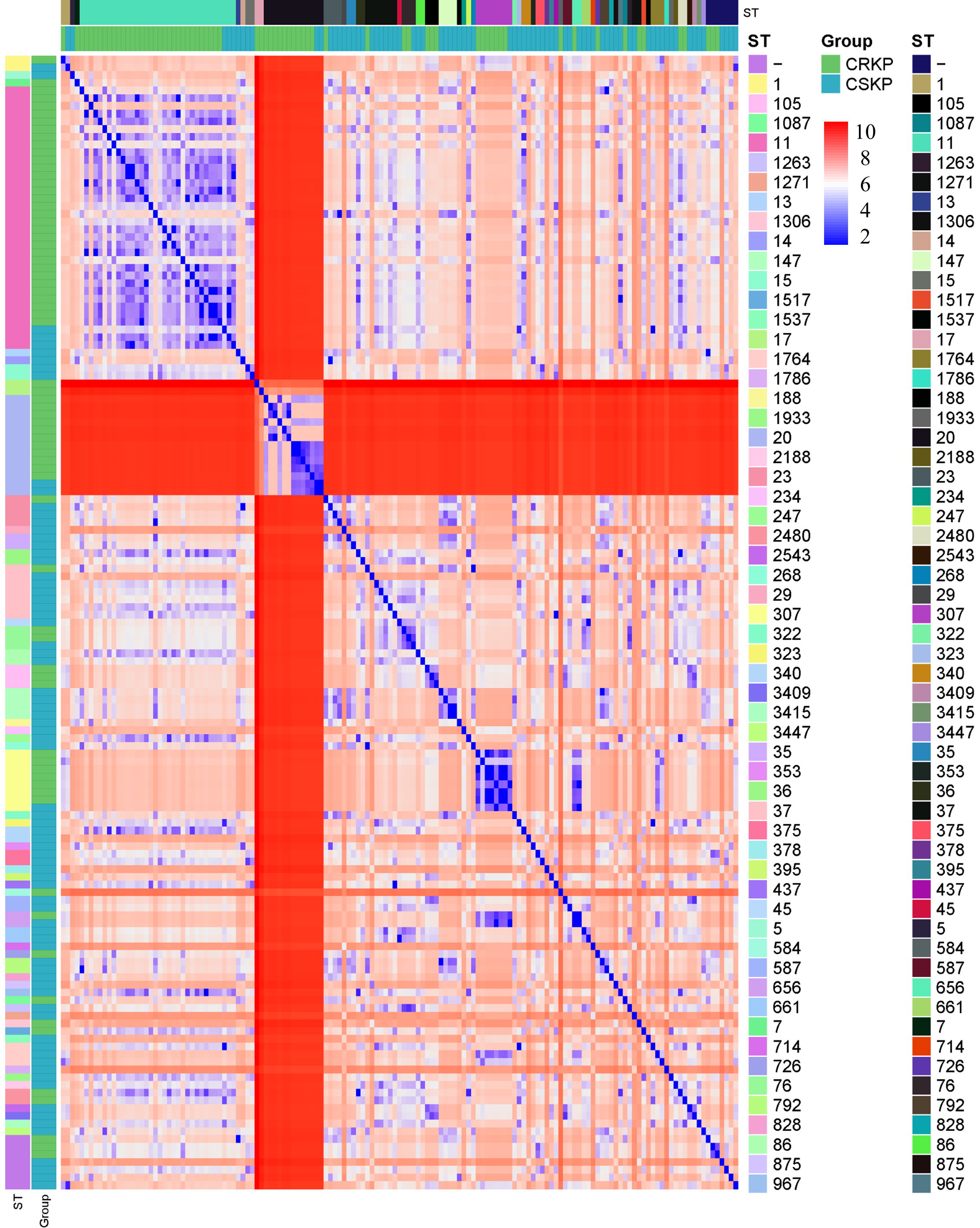

Supplement: Supplemental Material [file TEMI_A_1906163_SM8894.zip › Supplemental files/Figure S3.tif]

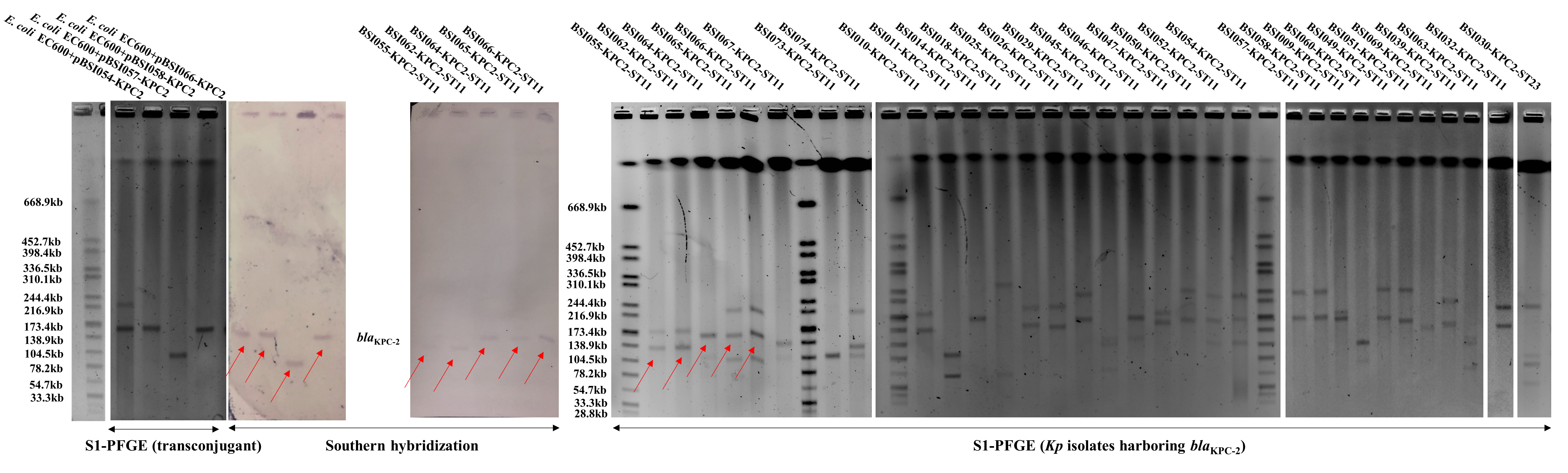

Supplement: Supplemental Material [file TEMI_A_1906163_SM8894.zip › Supplemental files/Figure S4.tif]

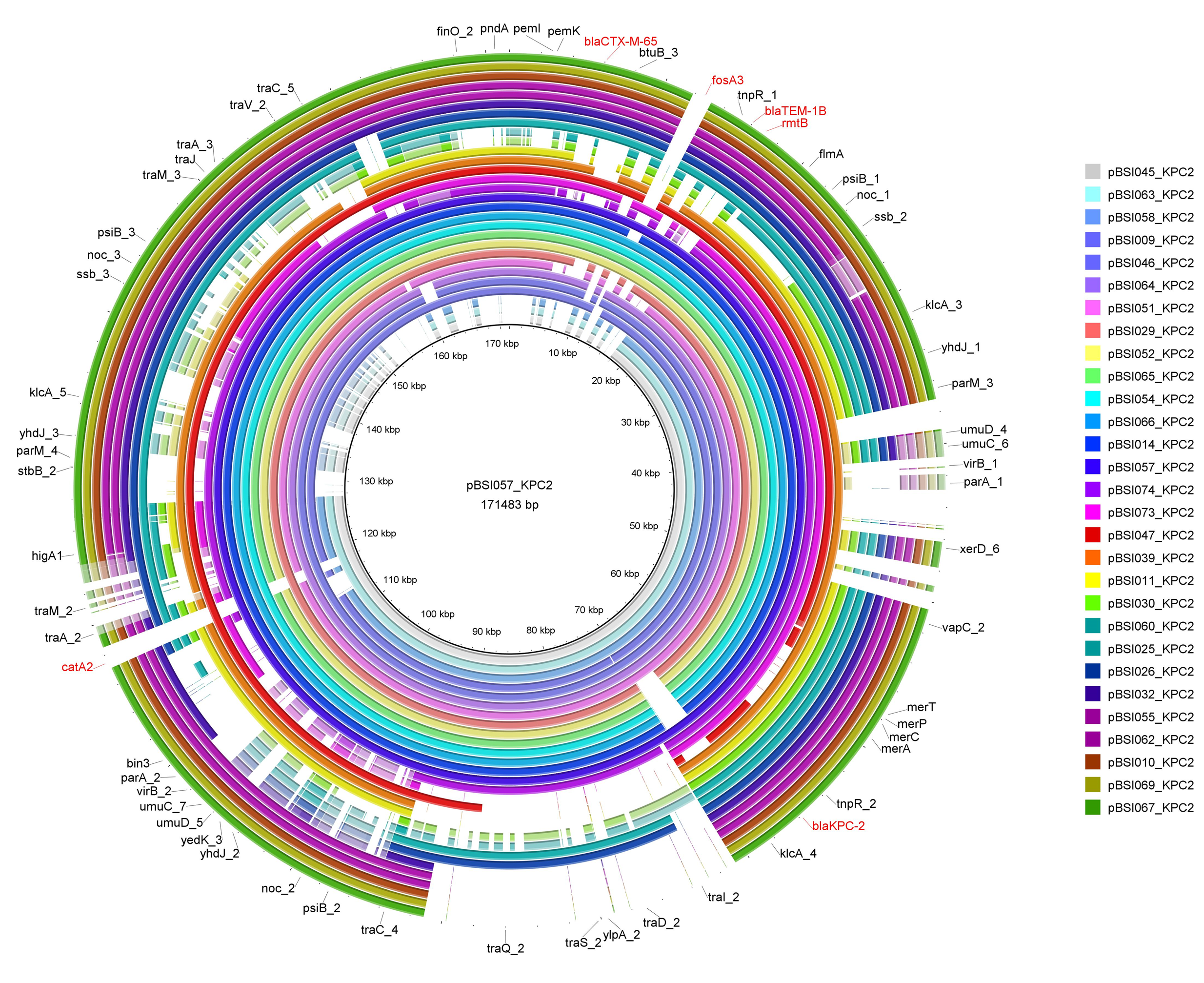

Supplement: Supplemental Material [file TEMI_A_1906163_SM8894.zip › Supplemental files/Figure S5.tif]
